# Characteristics of the Insulin-like Peptide Genes and Their Roles in the Ovarian Development of *Zeugodacus cucurbitae* (Coquillett)

**DOI:** 10.3390/insects16080854

**Published:** 2025-08-17

**Authors:** Jun-Chen Yi, Chuan-Lian Liu, Dong Chen, Dong Wei, Zhu-Ting Zhang

**Affiliations:** 1 Chongqing Key Laboratory of Entomology and Pest Control Engineering, College of Plant Protection, Southwest University, Chongqing 400715, China; m18623187421@163.com (J.-C.Y.); clliu23@163.com (C.-L.L.); cdyoucancd@email.swu.edu.cn (D.C.); 2 Key Laboratory of Agricultural Biosafety and Green Production of Upper Yangtze River (Ministry of Education), Southwest University, Chongqing 400715, China; 3 College of Life and Health Science, Kaili University, Kaili 556011, China

**Keywords:** melon fly, insulin-like peptide, nutrition stress, RNA interference, ovarian development

## Abstract

The melon fly *Zeugodacus cucurbitae* (Coquillett) is a globally invasive pest responsible for substantial economic losses in the fruit and vegetable industries. Insulin-like peptides (ILPs) are evolutionarily conserved neuropeptides that play a crucial role in insect reproduction. In this study, six *ZcILPs* from the melon fly, designated as *ZcILP1*–*6*, were cloned. Phylogenetic analysis demonstrated a strong orthologous link with Dipteran ILPs. Spatiotemporal expression profiling revealed that *ZcILP1* and *ZcILP3* exhibit preferential enrichment in the adult female fat body, with their expression specifically and significantly upregulated in 5-day-old individuals. Their expression decreased 12, 24, and 48 h post-starvation and increased upon re-feeding. Silencing *ZcILP1* and *ZcILP3* resulted in reduced ovarian size by 51.42% and 69.17%, respectively. Furthermore, silencing *ZcILP1* or *ZcILP3* significantly decreased the transcriptional levels of genes downstream of the insulin signaling pathway (ISP), notably the *target of rapamycin* (*ZcTOR*) and *Forkhead box O* (*ZcFOXO*). Concurrently, the expression of *Vitellogenin* (*ZcVg*), a gene associated with reproduction, was significantly downregulated. These findings indicate that *ZcILP1* and *ZcILP3* regulate *ZcVgs* expression and ovarian development through ISP, suggesting them as potential targets for green control of *Z. cucurbitae*.

## Simple Summary

Insulin-like peptides (ILPs), which function as hormones regulating essential physiological processes such as growth, development, and reproduction in insects, exhibit limitedly understood mechanisms of action in the reproductive regulation of agricultural pests. The melon fly, *Zeugodacus cucurbitae* (Coquillett), poses a significant threat to the secure global production of Cucurbitaceae crops. This study focuses on the biological functions of two ILP genes that are highly expressed in adult melon fly fat bodies, employing RNA interference (RNAi). Our results demonstrate that silencing these ILP genes significantly downregulates the transcription of key genes involved in ovarian development and the insulin signaling pathway (ISP). Furthermore, silencing the target genes led to decreased ovarian size in female flies, accompanied by a pronounced delay in developmental phenotype. This work highlights the critical role of these ILP genes in female melon fly reproduction, providing important molecular targets for developing novel RNAi-based pest control strategies.

## 1. Introduction

The melon fly, *Zeugodacus cucurbitae* Coquillett (Diptera: Tephritidae), is one of the globally recognized quarantine pests [1]. This pest causes damage to a wide range of vegetables and fruits, particularly those within the Cucurbitaceae, including cucumber, zucchini, luffa, bitter melon, pumpkin, and winter melon [2]. The primary damage is inflicted by the larvae of the melon fly, as female adults deposit their eggs within the fruit. The hatched larvae feed internally, causing initial yellowing in patches, followed by rotting and the release of a foul odor, which ultimately leads to a significant number of fallen fruits [3]. At present, the management of the melon fly primarily depends on chemical control methods [4]. Continuous reliance on chemical pesticides has caused melon flies to develop resistance over time [5]. Accordingly, imperative efforts are required to identify novel targets for formulating environmentally sustainable strategies against melon flies.

Insulin-like peptides (ILPs) are essential molecules in diverse organisms, exerting significant influence on various physiological processes such as cellular proliferation, metabolic regulation, and developmental pathways [6]. Bombyxin was the first ILP identified in insects, with subsequent discoveries revealing eight ILP genes in *Drosophila melanogaster* and *Aedes aegypti* [7,8,9]. Three ILP genes were identified in *Locusta migratoria*, whereas only one such gene was found in *Schistocerca gregaria* [10,11]. The structure and function of ILPs, along with the associated insulin signaling pathway (ISP), are highly conserved across biological species [12]. Invertebrates predominantly express ILPs, whereas vertebrates utilize a diverse array of related molecules, such as insulin, insulin-like growth factors, and relaxin, alongside ILPs [13]. The signaling pathway of ILPs has been extensively investigated in *D. melanogaster* [14].

Oogenesis is a complex phenomenon encompassing the previtellogenic, vitellogenic, and chorionic stages [15]. Among these, the vitellogenic phase is particularly energy-intensive, characterized by the synthesis and storage of essential nutrients, such as vitellin, lipids, and carbohydrates. These nutrients are synthesized across multiple tissues, secreted into the hemolymph, and subsequently incorporated by oocytes to facilitate egg production [16]. ILPs have been extensively studied and are recognized for their crucial function in regulating energy metabolism and nutrient homeostasis throughout the insect life cycle. They contribute to reducing the trehalose concentrations in the blood by promoting its conversion into glycogen for storage, thereby providing energy reserves during periods of food scarcity [11]. For instance, downregulation of the *NlILP1–3* expression has been demonstrated to increase glycogen levels in *Nilaparvata lugens* [13]. Conversely, ILPs bind to the insulin receptor (IR) and activate the phosphoinositide 3-kinase (PI3K)/protein kinase B (AKT) pathway. This activation accelerates glycolysis and facilitates the rapid conversion of hemolymph trehalose into energy for efficient utilization [17]. For instance, in *D. suzukii*, application of exogenous insulin led to a decrease in whole-body trehalose levels. Additionally, it significantly upregulated the expression of glycolytic enzymes such as hexokinase (Hk) and pyruvate kinase (Pk) [18].

Furthermore, the *target of rapamycin* (*TOR*) is closely integrated with the ISP, functioning as a regulatory checkpoint to ensure adequate nutrition for egg development [19,20]. These two pathways, which respond to nutrient availability and govern growth rates, are collectively termed the insulin/TOR signaling pathway. *Forkhead box O* (*FOXO*), a transcription factor, exerts a crucial influence on diverse cellular and physiological processes [21]. Previous research has demonstrated the close association of *FOXO* with nutritional pathways, particularly in regulating glucose homeostasis. For example, *BmFOXO* overexpression in *Bombyx mori* suppresses protein translation in the fat bodies, promotes glucose synthesis, and upregulates the expression of related genes. These effects ultimately lead to a reduction in hemolymph glycogen content [22].

Herein, six *ZcILPs* were identified from the *Z. cucurbitae* genome, and their spatiotemporal expression profiles were systematically analyzed across developmental stages and tissues. High expression of *ZcILP1* and *ZcILP3* was observed in the fat body, thereby prompting further exploration of their functions in ovarian development in *Z. cucurbitae*. A significant reduction in the gene expression associated with the nutritional signaling pathway, including *ZcTOR* and *ZcFOXO*, was observed following the knockdown of *ZcILP1* and *ZcILP3.* Additionally, the expression of reproductive-related gene, *Vitellogenin* (*ZcVgs*), was markedly decreased. These findings enhance our understanding of how ILPs regulate insect reproduction from a nutritional perspective.

## 2. Materials and Methods

### 2.1. Insects

In 2016, specimens of the melon fly *Z. cucurbitae* were gathered from Haikou, located in Hainan Province, and were subsequently maintained in a laboratory setting under regulated environmental parameters. The rearing temperature was maintained at 27.0 ± 0.5 °C, with a relative humidity of 70% ± 5%. The adults were reared at a photoperiod of 14 h:10 h (light/dark), whereas the larvae were maintained in complete darkness. The rearing methods for adults and larvae were followed as previously described [23].

### 2.2. RNA Isolation and cDNA Synthesis

To isolate total RNA, five-day-old virgin female adults were collected, and total RNA was extracted using TRIZOL reagent (Invitrogen, Carlsbad, CA, USA). Residual DNA contamination was then enzymatically removed using the RQ1 RNase-Free DNase kit (Promega, Madison, WI, USA). Subsequently, first-strand cDNA was synthesized using the PrimeScript^®^ RT Reagent Kit (TaKaRa, Dalian, China) and stored at –20 °C for long-term preservation and future applications.

### 2.3. Cloning and Molecular Sequence Analysis of Target Genes

Fragments of the *ZcILP1-6* were obtained from the genome of the melon fly. Primers were developed using the web-based tools (https://www.ncbi.nlm.nih.gov/tools/primer-blast/index.cgi?LINK_LOC=BlastHome, accessed on 22 January 2023) provided by the National Center for Biotechnology Information (NCBI) (Table S1). The open reading frame (ORF) sequences of *ZcILPs* were cloned using cDNA derived from five-day-old melon fly adults. Phylogenetic analysis was conducted using the neighbor-joining algorithm in MEGA-X (Auckland, New Zealand), with 1000 bootstrap replicates incorporated to assess clade robustness. Sequence alignments were performed across the obtained datasets using Jalview (V2.11; Dundee, UK).

### 2.4. Spatio-Temporal Expression Patterns Analysis

Samples from various developmental stages, including eggs, 1st, 2nd, and 3rd instar larvae, 1-, 5-, and 9-day-old pupae, and 1-, 5-, and 9-day-old virgin male and female adults, were collected, with three biological replicates per stage. Tissue samples, including the midgut, Malpighian tubules, fat body, ovaries, and testes, were dissected from 5-day-old adult flies. The primers were designed using the NCBI online tool (Table S1).

### 2.5. Gene Expression Analysis Induced by Starvation

A total of 120 three-day-old female flies were collected and assigned to three experimental groups. The first group (*n* = 40) was fed on a normal diet, while the second group (*n* = 40) was provided with water only. Female flies from these two groups were collected at 12, 24, and 48 h for further analysis. To analyze the effect of refeeding on gene expression, a third group (*n* = 40) underwent a 24 h starvation period (water only), followed by 24 h of feeding with a normal diet. Samples from this group were collected 24 h after refeeding, corresponding to 48 h from the start of the experiment. For the analysis of *ZcILPs* expression dynamics under nutritional stress conditions, *Rps3* and *RpL13* were meticulously chosen as internal reference genes to normalize transcriptional data [24].

### 2.6. RNA Interference (RNAi) and Functional Analysis

Prepared in total, 180 three-day-old female melon flies were randomly assigned to three experimental groups with 60 females in each group. Each group received two injections of *dsZcILP1*, *dsZcILP3*, or *dsGFP* at a 24 h interval over two consecutive days. The dsRNA dose for injection was 2 µg, and the injections were carried out using a Micromanipulator M3301R (World Precision Instruments, Sarasota, FL, USA). Samples were collected 24 h after the second injection for the total RNA isolated from a pooled sample of three individuals per replicate. Three biological replicates were prepared for the RNA isolation. *α-tub* and *Rps3* were selected as references for RT-qPCR to assess the silencing efficiency [25].

A systematic analysis of transcriptional abundances was conducted for key downstream genes involved in insect reproductive biology, such as *ZcTOR*, *ZcFOXO*, and *ZcVgs*, with the aim of characterizing their expression patterns. The ovaries in each group were dissected from 20 virgin females. 

### 2.7. Statistical Analysis

Student’s *t*-test was used to evaluate the effects of nutritional stress on gene expression profiles, gene silencing efficiency, expression levels of pathway genes, and ovarian size measurements. Ovarian size was quantified using the Leica Application Suite X (V3.7) software under a Leica M205A stereomicroscope (Wetzlar, Germany). The normality of all data was evaluated using the Shapiro–Wilk test. For the ovarian size measurement experiment, 20 biological replicates were performed, while three biological and two technical replicates were adopted for the remaining experiments. Statistical analyses were performed using the Statistical Package for Social Sciences (SPSS) software (V22.0; IBM, Armonk, NY, USA).

## 3. Results

### 3.1. Sequence Analysis and Phylogenetic Comparison

**Figure 1:**
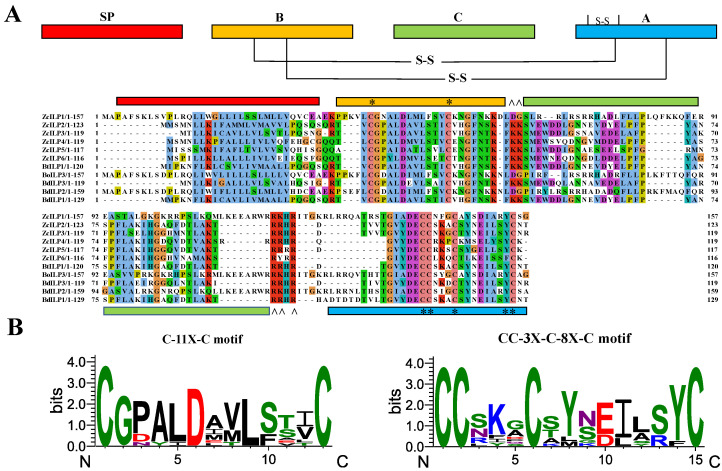
Sequence analysis of ILPs in *Zeugodacus cucurbitae* and other dipteran insects. (**A**) ZcILP1-6 consists of a signal peptide (red), B-chain (yellow), C-chain (green), and A-chain (blue) arranged from the N-terminus. The asterisk (*) denotes cysteine residues, and the caret (^) indicates predicted cleavage sites. (**B**) The WebLogo illustrates the conservation of amino acid sequences at C-11X-C and CC-3X-C-8X-C sites. *Z. cucurbitae*, Zc; *Bactrocera oleae*, Bo; *B. dorsalis*, Bd; *B. tryoni*, Bt.

Six ILP genes were uncovered through comprehensive screening of the *Z. cucurbitae* genomic sequence: XM_054229369.1, XM_011185342.3, XM_011185345.3, XM_029040709.2, XM_011185346.3, and XM_029040710.2. PCR validated the ORF with the sizes of 480, 372, 372, 360, 354, and 351 bp, encoding proteins of 160, 124, 124, 120, 118, and 117 amino acids, respectively. The molecular weights of the identified ZcILP proteins were 18.14, 13.94, 13.69, 13.64, 12.98, and 13.19 kDa, with corresponding isoelectric points of 11.01, 6.74, 6.88, 8.04, 7.77, and 5.72, respectively. Structurally, ZcILPs consist of a B-C-A tripeptide chain along with an N-terminal signal peptide (Figure 1A). They exhibit highly conserved structural features, including C-11X-C and CC-3X-C-8X-C, present in B and A chains, respectively. A and B chains are interconnected by disulfide bonds, which are formed both within and between chains. Additionally, the number and location of cysteine residues in ZcILPs are highly conserved (Figure 1B).

**Figure 2:**
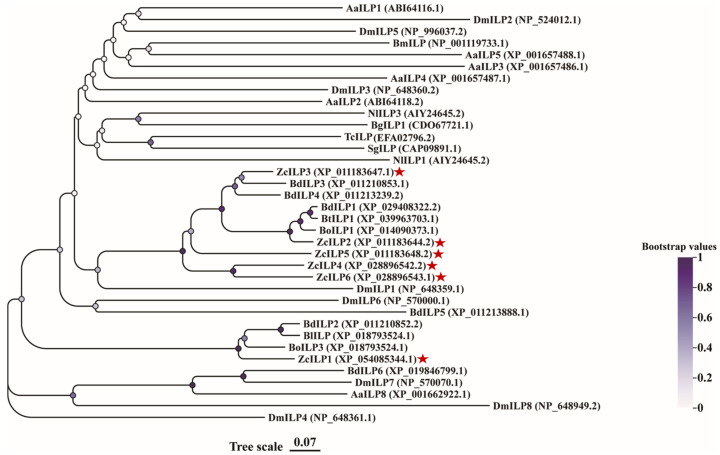
Phylogenetic tree of melon fruit fly and other insects based on ILPs. The ZcILP1-6 is marked by a red triangle. Aa, *Aedes aegypti*; Dm, *Drosophila melanogaster*; Nl, *Nilaparvata lugens*; Bg, *Blattella germanica*; Tc, *Tribolium castaneum*; Sg, *Schistocerca gregaria*; Bd, *Bactrocera dorsalis*; Bt, *B. tryoni*; Bo, *B. oleae*; Bl, *B. latifrons*. The accession numbers for each amino acid sequence are provided at the end of the corresponding proteins.

The phylogenetic analysis revealed that the amino acid sequences of ZcILPs cluster together with those of *Bactrocera dorsalis* and *B. oleae*. As all three species belong to the family Tephritidae, their ILPs exhibit high homology, while their affinity to the orders Lepidoptera and Coleoptera is more distant (Figure 2).

### 3.2. Spatio-Temporal Expression of the Insulin-like Peptide Genes

**Figure 3:**
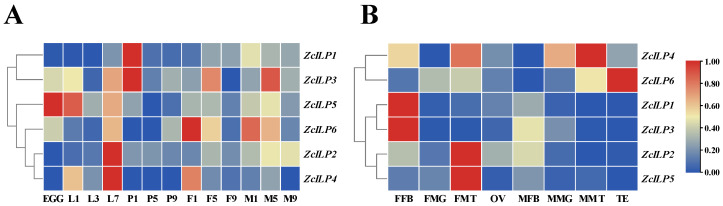
Spatio-temporal expression pattern of six *ZcILPs*. (**A**) Expression patterns of *ZcILP1-6* across different developmental stages. L1, L3, L7: 1-, 3-, and 7-day-old larvae; P1, P5, P9: 1-, 5-, and 9-day-old pupae; F1, F5, F9: 1-, 5-, and 9-day-old females; M1, M5, M9: 1-, 5-, and 9-day-old males. (**B**) Expression of *ZcILPs* in the tissues of 5-day-old males and females. FB: fat body, MG: midgut, MT: Malpighian tubule, OV: ovary, TE: testis.

*ZcILP1* and *ZcILP3* exhibited high expression levels on the first day of the pupal stage and in the adult stage, whereas *ZcILP2* demonstrated peak expression during the 3rd-instar larval stage. In contrast, the expression levels of *ZcILP4*, *ZcILP5*, and *ZcILP6* genes remained relatively low across all developmental stages (Figure 3A). The expression patterns of *ZcILPs* varied across different tissues of *Z. cucurbitae*. *ZcILP1* and *ZcILP3* were highly expressed in the female fat body, whereas *ZcILP2* and *ZcILP5* exhibited predominant expression in the female Malpighian tubules. *ZcILP4* demonstrated elevated expression in the male midgut as well as in both male and female Malpighian tubules, reaching its peak in the male Malpighian tubule. Furthermore, *ZcILP6* exhibited the highest transcript levels in the testis (Figure 3B).

### 3.3. Expression of ZcILPs Induced by Starvation

**Figure 4:**
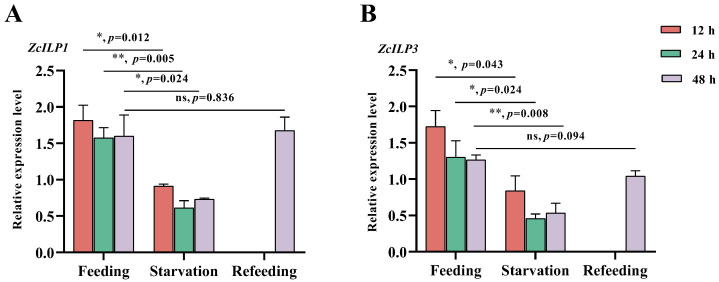
Effect of nutritional stress on *ZcILP1* and *ZcILP3* expression. (**A**) Expression levels of *ZcILP1* were analyzed following 12, 24, and 48 h of starvation, as well as after 24 h of starvation, followed by 24 h of feeding. (**B**) Expression levels of *ZcILP3* were assessed under the same conditions. The bars represent the mean ± standard error of the mean (SEM) derived from three biological replicates. Asterisks indicate significant differences determined by a Student’s *t*-test, ns indicates no significant difference.

*ZcILP1* and *ZcILP3* were highly expressed in the adult female fat body; accordingly, their expression was analyzed during starvation. The results demonstrated a significant reduction in *ZcILP1* expression, with decreases of 50.0%, 61.1%, and 56.5% observed after 12, 24, and 48 h of starvation, respectively (Figure 4A). Furthermore, *ZcILP1* expression following 24 h of starvation and a subsequent 24 h feeding period was comparable to that observed in normally fed flies. In contrast, *ZcILP3* expression decreased by 24.0%, 64.9%, and 53.8% after 12, 24, and 48 h of starvation, respectively (Figure 4B). Notably, after 24 h of starvation followed by 24 h of feeding, *ZcILP3* expression was restored to normal levels.

### 3.4. Effect of RNAi on Adult Reproduction of Z. cucurbitae

**Figure 5:**
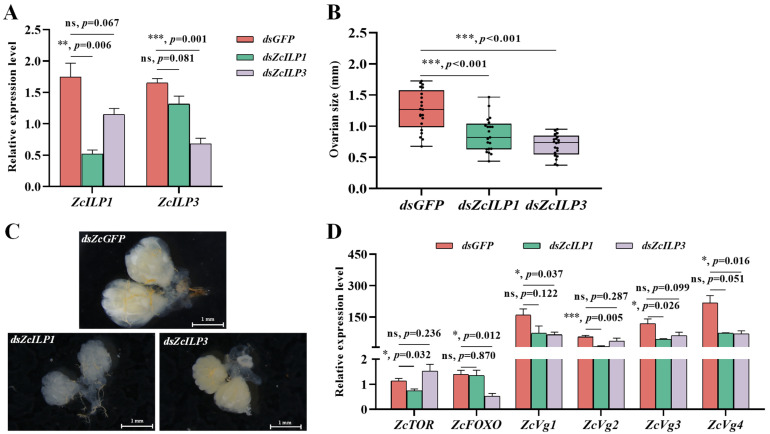
Impact of *ZcILP1* and *ZcILP3* suppressing on ovarian development and the expression of downstream genes in female *Zeugodacus cucurbitae*. (**A**) The silencing efficacy of *ZcILP1* and *ZcILP3* was assessed 24 h post-injection (*n* = 3). (**B**) The ovarian diameter of 5-day-old female melon flies was measured following injections with *dsGFP*, *dsZcILP1*, and *dsZcILP3* (*n* = 20). (**C**) Representative images of ovaries from 5-day-old female *Z. cucurbitae* following RNA interference (RNAi) treatment. (**D**) The expression levels of *ZcTOR*, *ZcFOXO*, and *ZcVgs* were evaluated post-injection with *dsGFP*, *dsZcILP1*, and *dsZcILP3* (*n* = 3). Data are presented as mean ± SEM from three biological replicates. Asterisks indicate significant differences determined by a Student’s *t*-test, ns indicates no significant difference.

The expression levels of *ZcILP1* and *ZcILP3* were reduced by 70.3% and 56.8%, respectively, following treatment with gene-specific dsRNA (Figure 5A). No cross-interference was detected following the processing of *dsZcILP1* and *dsZcILP3*. Moreover, the ovarian size of female adults in *dsZcILP1* and *dsZcILP3* treatment groups was significantly smaller compared to the *dsGFP* control group (Figure 5B,C). *ZcTOR* expression decreased after *dsZcILP1* treatment but remained unchanged after *dsZcILP3* silencing. Conversely, *ZcFOXO* expression was unaffected by *dsZcILP1* treatment but decreased by 62.59% following *dsZcILP3* silencing (Figure 5D). Moreover, the expression levels of *ZcVg2* and *ZcVg3* decreased, while the expression levels of *ZcVg1* and *ZcVg4* remained relatively unchanged after *dsZcILP1* treatment. *ZcVg1* and *ZcVg4* were significantly downregulated, whereas the expression levels of *ZcVg2* and *ZcVg3* did not exhibit significant changes following *dsZcILP3* treatment (Figure 5D).

## 4. Discussion

ILPs constitute a well-characterized group of peptide hormones. The signaling pathways associated with ILPs involve receptor proteins and downstream components, demonstrating a high degree of conservation across metazoans [26]. ILPs have been identified to regulate a variety of metabolic processes, including growth, development, reproduction, and lifespan regulation in insects [27]. In this study, six ILP genes were identified in *Z. cucurbitae*. The number of ILP genes varies among species. For instance, *D. melanogaster* possesses eight ILPs, *B. dorsalis* has six, and *Apis mellifera* contains only two [28,29,30]. The variation in the number of ILP genes among insects may reflect their potential evolutionary adaptations and selection strategies [31]. Results from multiple sequence alignments of ILPs in the melon fly reveal that these *ZcILPs* exhibit typical structural characteristics of ILPs.

The diversity in ILP gene expression has been observed in various insect species. In *D. melanogaster*, *DmILP1* exhibited widespread expression in brain tissue, encompassing larval and adult stages. *DmILP3* was predominantly expressed in the adult midgut, whereas *DmILP4* was restricted to the larval midgut. *DmILP5* displayed expression in adult ovaries and Malpighian tubules, while *DmILP6* was significantly expressed in the adipose tissues of both larvae and adults. Furthermore, *DmILP8* was primarily localized in adult imaginal discs and ovaries [9,32]. Moreover, in *A. aegypti*, *AaILP5* and *AaILP6* were localized to the fat body, epidermis, and abdominal nerve cord, with *AaILP2* exhibiting high expression exclusively in the ovaries [33]. The temporal variation in *ZcILP* expression suggests functional diversity among these peptides. For instance, *DmILP6* is involved in regulating carbohydrate and fat metabolism and modulates antioxidant defense mechanisms [32]. Similarly, *DmILP8*, which is highly expressed in the ovaries, has been implicated in regulating female reproductive capacity [34]. However, in this study, no ovary-specific ILP was identified in the melon fly. It is well established that ILP secretion and function in insects are closely associated with nutritional status, with the fat body playing a central role in energy storage and utilization. The fat body serves as the central metabolic organ in insects, playing a pivotal role in energy storage, particularly glycogen and lipid reserves, and in regulating metabolic processes. It is essential for maintaining energy homeostasis and ensuring proper metabolic function in these organisms [35]. The metabolism of fats and carbohydrates, along with protein synthesis, predominantly occurs within the fat body in most insects [36]. Considering the critical role of nutrient stores in the fat body for supporting ovarian development and egg production, the elevated expression of *ZcILP1* and *ZcILP3* in the fat bodies of female adults suggests a role in nutrient sensing during ovarian development, while the remaining four ILP genes (*ZcILP2*, *ZcILP4–6*) may participate in distinct aspects of melon fly physiology. *ZcILP2*, *ZcILP4*, and *ZcILP5* are highly expressed in the midgut, so these peptides may regulate midgut cell growth, analogous to *DILP3* in *D. melanogaster*, which directly modulates intestinal stem cell proliferation [37]. By contrast, *ZcILP6* is predominantly expressed in the adult male testis, suggesting a possible role in spermatogenesis. However, functional studies of testis-specific ILPs in insects remain scarce, and their precise roles warrant further investigation.

Nutrition is currently recognized as the most critical factor regulating ILP secretion, which, in turn, influences the transduction of ISP [11]. Following nutrient intake, the fat body rapidly detects these nutrients and responds by secreting ILPs. This process also involves the transmission of nutritional signals to the brain, which subsequently regulates ILP synthesis [38,39]. ILPs play a fundamental role in regulating carbohydrate levels, which serve as the primary energy source for insects. This regulation, in turn, influences their growth, development, and reproductive processes, thereby contributing to the maintenance of homeostasis. Notably, starvation has been demonstrated to significantly reduce the expression of *BgILPs* in *Blattella germanica* [40]. The expression levels of *BtILP1-3* in *Bemisia tabaci* were significantly downregulated after 12 h of starvation; however, they were restored within 1 h of feeding on an artificial diet [41]. Within our investigation, the transcriptional levels of *ZcILP1* and *ZcILP3* were downregulated under starvation conditions and subsequently recovered upon subsequent refeeding. These findings provide compelling evidence that the highly expressed *ZcILP1* and *ZcILP3* in the fat body function as nutritional sensors in *Z. cucurbitae*, detecting changes in internal nutrient levels. The role of ILPs as nutrient sensors has been extensively studied across various insect species.

ILPs are well-established as key regulators of female reproductive mechanisms across a wide range of insect species. For instance, ILP3 injection stimulates egg-associated haemocyte proliferation in *A. aegypti* [42]. The administration of bovine insulin has been reported to enhance ovarian growth and improve fertility in *Chrysopa pallens* [43]. Conversely, the silencing of *CpILP1* and *CpILP2* has been observed to significantly inhibit ovarian growth and severely disrupt ovarian morphology [44]. In the present study, suppression of *ZcILP1* and *ZcILP3* expression resulted in delayed ovarian development in *Z. cucurbitae*, accompanied by varying degrees of down-regulation in *ZcVgs*. These findings strongly suggest that *ZcILP1* and *ZcILP3* play a regulatory role in female melon fly reproduction and function upstream of *ZcVgs*. So, these critical genes may be a potential target for pest control in the next generation of pesticide development, although there is still a huge challenge to overcome. Future applications could combine dsRNA with nanocarriers, which protect against nucleases and alkaline hydrolysis to overcome RNAi barriers [45]. Studies on Hemiptera, Coleoptera, and Lepidoptera have demonstrated successful RNAi via transgenic plants; for example, chloroplast-expressed β-actin dsRNA in potato causes 100% mortality in *Leptinotarsa decemlineata* [46,47].

ISP is a critical nutritional signaling mechanism in insects, with ILPs serving as upstream sensors of nutritional signals. *TOR* and *FOXO* play crucial roles in nutritional regulation downstream of ISP [48]. *TOR* is a vital cellular regulatory factor that interacts with other proteins to regulate essential processes, including energy metabolism and protein synthesis in insects [49]. *ZcILP1* knockdown in our investigation resulted in a notable decrease in *ZcTOR* expression, highlighting its regulatory influence in this context. Similar findings have been reported in the *Diaphorina citri*, indicating that lower *DcILP* levels downregulate *DcRheb* expression in the TOR pathway, significantly affecting reproduction in *D. citri* [50]. In fact, the role of *TOR* in reproduction is mediated through nutritional signals, which subsequently influence the titers of 20-hydroxyecdysone (20E) and juvenile hormone (JH) in adult insects [51]. For instance, in *B. germanica*, *BgTOR* suppression resulted in significant inhibition of JH synthesis in female adults, leading to impaired ovarian development [52]. The findings indicate that *ZcILP1* acts as an upstream regulatory factor, influencing downstream reproductive functions by perceiving nutritional signals, potentially through the TOR signaling pathway. Silencing *ZcILP1* significantly downregulates *ZcTOR* transcription, via the canonical IR–PI3K–Akt cascade. In *Drosophila*, ILP binding to IR sequentially activates PI3K and Akt, which phosphorylate and thereby activate TOR [48]. Conversely, TOR can also be activated through direct nutrient sensing, indicating that its regulation is multifaceted [53]. Further experiments are required to elucidate the precise mechanism underlying this regulation.

FOXO is an evolutionarily conserved key transcription factor acting downstream of the ISP, with its activity modulated by this pathway [54]. In this study, specific interference with *ZcILP3* resulted in a significant downregulation of *ZcFOXO* expression. Similarly, *LmFOXO* has been reported to regulate insect reproduction by activating *Vg* expression and promoting egg maturation in *L. migratoria* [55]. In *Chrysoperla sinica*, *CsFOXO* has also been implicated in ovarian development, whereas its suppression results in downregulated transcription of the vitellogenin gene (*CsVg*) and a subsequent decline in oviposition capacity [26]. Accordingly, *ZcILP3* may regulate the transcription of *ZcVgs* via the transcription factor *ZcFOXO* within ISP, consequently modulating the reproductive functions of the melon fly.

## 5. Conclusions

In the current investigation, six *ZcILP* genes were successfully isolated and characterized from the genome of the melon fly. The spatio-temporal expression analysis demonstrated that among the six *ZcILP* genes, only *ZcILP1* and *ZcILP3* were predominantly expressed in the fat body, with their expression levels decreasing under starvation conditions. Silencing these two genes led to delayed ovarian development. We proposed that nutrient availability influences the reproduction of melon flies through ISP activation. This process involves the generation of nutritional signals upon food intake, which are transmitted to the *TOR* and *FOXO* pathways via *ZcILP1* and *ZcILP3*, respectively. The activation of *TOR* and *FOXO* subsequently upregulates *Vg* expression, thereby facilitating ovarian development. Although RNAi assays demonstrated that knockdown of *ZcILP1* and *ZcILP3* significantly alters the transcript levels of *ZcTOR*, *ZcFOXO*, and *ZcVgs*, corresponding protein-level validation was not performed. Consequently, whether these genes are similarly regulated at the translational or post-translational level remains to be determined.

## Data Availability

The original contributions presented in this study are included in the article/Supplementary Materials. Further inquiries can be directed to the corresponding authors.

## Supplementary Materials

The following supporting information can be downloaded at https://www.mdpi.com/article/10.3390/insects16080854/s1, Table S1. Primers used in this study.
