# Supplementary material for: Characteristics of the Insulin-like Peptide Genes and Their Roles in the Ovarian Development of Zeugodacus cucurbitae (Coquillett)
Source: Insects. 2025 Aug 17;16(8):854. doi: 10.3390/insects16080854 (PMC12386508; doi:10.3390/insects16080854)
Supplement: Supplementary file 1 [file insects-16-00854-s001.zip › insects-3763979-supplementary.pdf]

Table S1. Primers used in this study.

Name	Upstream primers (5′-3′)	Downstream primers (5′-3′)	Application
ZcILP1	ATAGCGGTTCGAGAGGTAAA	CTTCTACTTTGGGTCTCAGC	Clone
ZcILP2	AGCAGCTTTTTAGGCGTTCC	ACATGTTTCTCCATCCTGGGC	
ZcILP3	TGTTAGCATATTAATCCTGGTTT	TCTATTAAAACCGCAACA	
ZcILP4	AGTTGCCTTACACGCCTAAA	TTTGTCAGTTTGTGGGGAGC	
ZcILP5	AACAGAACGTTGAGTGGAAAT	CTACTTAAGAGGCTGCCTGA	
ZcILP6	AGCATCGGGTTCATTAGCCA	CATTTGTTGCTGAGAGCGCA	
qZcILP1	AAAGGTAAGCGTCGTCCCAG	AAAGTTGCAGCATTCGTCGG	qPCR
qZcILP2	GTGGGACGATCTGGGAAACA	ATTGGGCGCCATGGATCTTT	
qZcILP3	ATGGCAGGACCGTTTGTGGA	CCACAGCATTGCTACCCAGG	
qZcILP4	AGCAAACTCTCTGTGGTCCC	CGGCAGCATTCATCATACAC	
qZcILP5	CGAAAACGGCTTTAACACGA	GTACACACCATCACGACGAC	
qZcILP6	TGGCCAACAAACTATCTGCG	GGAAAGGGAAGCCTGCATAA	
qZcVg1	GCCAAATGATCGGCAAGACC	GAAGATCTTTGCGGGGTCCA	
qZcVg2	TCTAGCCGTTCAAGCAGTCA	GTGTTGCCGGTTTGACG	
qZcVg3	GCTCCACACTCACCAACATG	CGAACATCTTGGCAGGGT	
qZcVg4	CACTCACCAACATGAAGCGT	TTAGCAGGGTCCAAAGC	
qZcTOR	ACCGATCCATCCACACAACC	CGCCTGTACCACCTTGAAGT	
qZcFOXO	CACCGCCCACAGATATACCC	ATACGGCACATTCTGCACCA	
αTub	CGCATTCATGGTTGATAACG	GGGCACCAAGTTAGTCTGGA	
Rps3	TAAGTTGACCGGAGGTTTGG	TGGATCACCAGAGTGGATCA	
βTub1	GAATTGATGCGACTGGTGCC	CTGAATCCATGGTGCCAGGT	
Rpl13	GTTGTGCGTTGCGAGGAATT	GCTTGTCGTATGGTGGTGGA	
dsZcILP1	TaatacgactcactatagggAACGCTCTGGAC CTTATGCT	TaatacgactcactatagggAGTGCTGT CGATGCTTCCC	RNAi
dsZcILP3	TaatacgactcactatagggTCACCTCAAACC GATTGCAGTA	TaatacgactcactatagggCCGTTCCT ATCCGAGCTTCA	
dsGFP	TaatacgactcactatagggTGAGCAAGGGCG AGGAGCTG	TaatacgactcactatagggTCGATGCG GTTCACCAG	
